# Critical Role of the Posterior Left Atrium in the Perpetuation of Persistent Atrial Fibrillation and the Hybrid Ablation Approach for Persistent Atrial Fibrillation Management: A Single-center Outcomes Study

**DOI:** 10.19102/icrm.2018.091003

**Published:** 2018-10-15

**Authors:** Rangadham Nagarakanti, Keung Ung, Hillary Strahan

**Affiliations:** ^1^Department of Cardiology, Owensboro Health Regional Hospital, Owensboro, KY, USA; ^2^Rutgers Robert Wood Johnson Medical School, Piscataway, NJ, USA

**Keywords:** Catheter ablation, hybrid atrial fibrillation ablation, persistent atrial fibrillation, posterior left atrium, pulmonary vein region

## Abstract

Atrial fibrillation (AF) is the most common heart rhythm disorder and a growing major public health burden. AF ablation is considered to be the preferred rhythm control strategy for symptomatic drug-refractory paroxysmal and persistent AF (PRAF). To date, the long-term ablation success rates of pulmonary vein isolation (PVI) for PRAF and longstanding PRAF (LS-PRAF) have not paralleled those of paroxysmal AF. Additional concomitant ablation strategies such as linear ablation lesions in the left and right atria; autonomic ganglionic plexi ablation; ablation directed by complex fractionated atrial electrograms; ablation of nonpulmonary vein (PV) triggers; radiofrequency ablation of the vein of Marshall; and, most recently, focal impulse and rotor ablation/modulation have shown modest improvement in terms of efficacy, but no reproducible outcomes. Here, we describe the critical role of the posterior left atrium (LA) and PV region in the development and progression of PRAF and LS-PRAF. We discuss the results of single-center outcomes data for convergent or hybrid AF ablation of the posterior LA and PV region (endocardial PVI + minimally invasive epicardial posterior LA ablation). This epicardial ablation approach, combined with endocardial ablation, is an option for patients with PRAF and LS-PRAF. More definitive clinical trials are needed.

## Introduction

Atrial fibrillation (AF) is a growing major public health burden with an increasing prevalence. Ablation is typically recommended for drug-resistant, symptomatic paroxysmal and persistent AF (PRAF).^1^ The pathophysiological mechanisms of AF are complex, requiring both the triggers and the presence of predisposing substrate(s) for the triggering and perpetuation of AF. Several investigators have shown that arrhythmogenic activity in the pulmonary veins (PVs) triggers and/or perpetuates episodes of AF.^2,3^ Therefore, PV isolation (PVI) as a sole strategy is considered to be a cornerstone for the nonpharmacologic management of paroxysmal AF. However, the best nonpharmacologic approach for the treatment of PRAF and longstanding PRAF (LS-PRAF) remains under discussion. The substrate is a key element in the progression of paroxysmal AF to PRAF and LS-PRAF. PVI, including wide-area antral isolation alone, has been shown to be insufficient and to result in less favorable outcomes in patients with PRAF and LS-PRAF.

The mechanisms for the development and progression of PRAF and LS-PRAF are still not completely understood, and this has limited the creation of effective management strategies. To date, there have been several proposed mechanisms for the onset and worsening of AF, such as ganglionated plexi, non-PV triggers, complex fractionated atrial electrograms (CFAEs), and macroreentrant mechanisms such as rotors. However, the additional therapeutic ablation strategies **(Table 1)** employed in light of these proposed mechanisms along with PVI only have minimal to modest success, as reflected by several clinical trials and meta-analyses.^4–6^

The three-year arrhythmia-free survival rate in patients with PRAF following a single endocardial ablation procedure has been reported to be 28.4%, with the efficacy after multiple procedures being 51.1%.^4^ The Hamburg sequential ablation strategy of PVI plus linear ablation lesions and superior vena cava isolation for patients with LS-PRAF yielded five-year single- and multiple-procedure success rates of 20% and 45%, respectively.^5^ Recently, the Substrate and Trigger Ablation for Reduction of AF Trial Part II (STAR AF II) study demonstrated that PVI-only ablation in patients with PRAF resulted in a single-procedure freedom from atrial tachyarrhythmias off antiarrhythmic drugs rate of 59% at 18 months.^6^ The addition of CFAE ablation or linear ablation lesions actually yielded even lower rates of 48% and 44% for freedom from atrial tachyarrhythmias, respectively.^6^ Ablation of ganglionated plexi alone in patients with LS-PRAF, however, led to poorer results, with a rate of maintenance of sinus rhythm of 38.2% at 24 months after surgery.^7^ Repeat procedures with circumferential PVI yielded a success rate of 59.6% over a follow-up of 16 months ± seven months.^7^ The focal impulse and rotor modulation “rotor” ablation technique also showed poor long-term efficacy in this patient cohort, with only 21% being free of recurrent AF and only 12% being free of AF and off antiarrhythmic drugs at 16.0 months ± 10.7 months (range: 1–34 months) follow-up.^8^

Here, we describe our technique and the single-center outcomes data for convergent or hybrid AF ablation of the PV region (staged minimally invasive epicardial posterior LA ablation + endocardial PVI) for the management of PRAF and LS-PRAF.

## Embryology of the posterior left atrium

**Figure 1:**
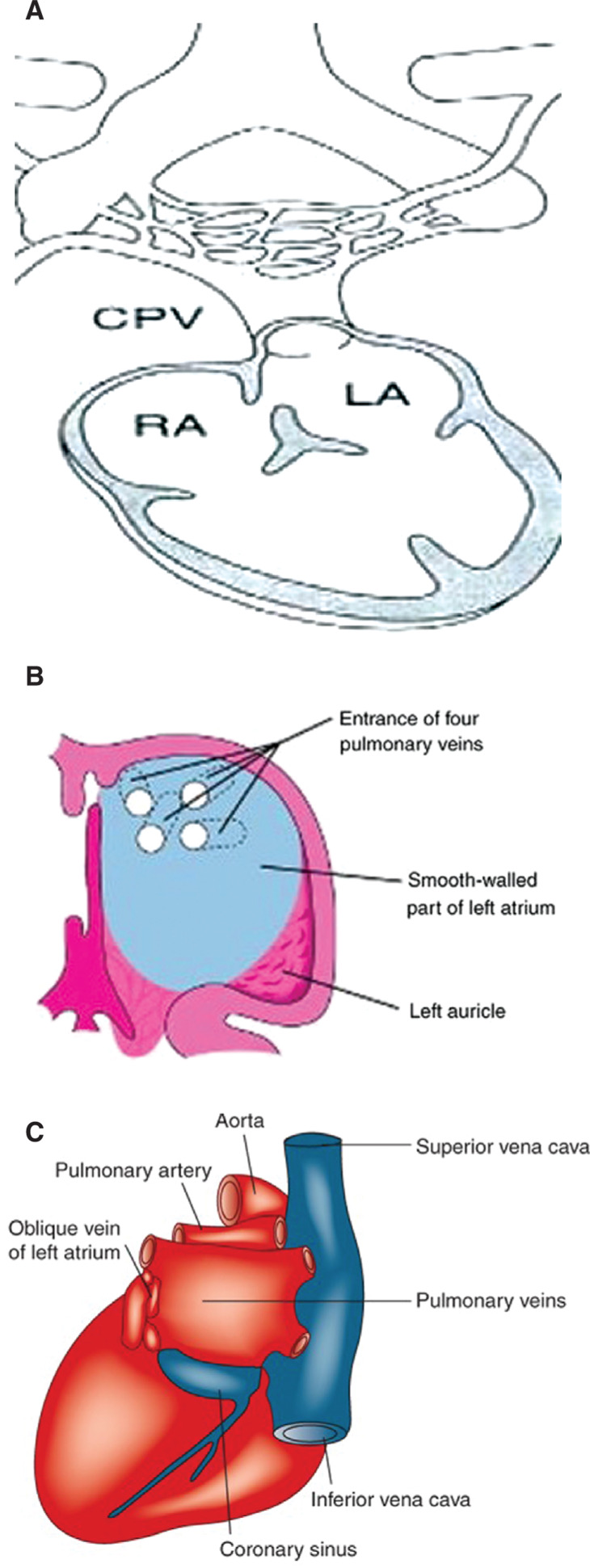
Embryology of the posterior LA and PV region. **A:** The common PV connects to the posterior LA in utero. CPV: common pulmonary vein; LA: left atrium; RA: right atrium. **B:** The smooth-walled posterior LA is derived from absorbed PV tissue. The left auricle (LA appendage) is derived from the primitive atrium. **C:** Posterior external anatomy of the heart with PVs connected to the LA.

During fetal development, the PVs initially have no connection to the embryologic heart. They then converge to form the common PV (at about the fifth or sixth month in utero) that becomes incorporated into the left atrium (LA) by connecting to the embryologic LA on the posterior aspect. The convergence of these PVs forms the smooth posterior LA. After incorporation, the composition of the PVs and the smooth-walled posterior LA body wall are histologically identical **(Figures 1A–1C)**.^9^

In addition, the embyologic primitive atria become the LA appendage. The LA appendage is histologically different from the PVs and consists of endocardial and myocardial layers without a vessel wall component. The posterior LA remains the receiving chamber throughout adult life and has no significant mechanical or contractile function.

## Role of the posterior left atrium and pulmonary vein region in the progression of persistent atrial fibrillation and longstanding persistent atrial fibrillation

### Anatomical and mechanical changes in the posterior left atrium with the progression of atrial fibrillation

**Figure 2:**
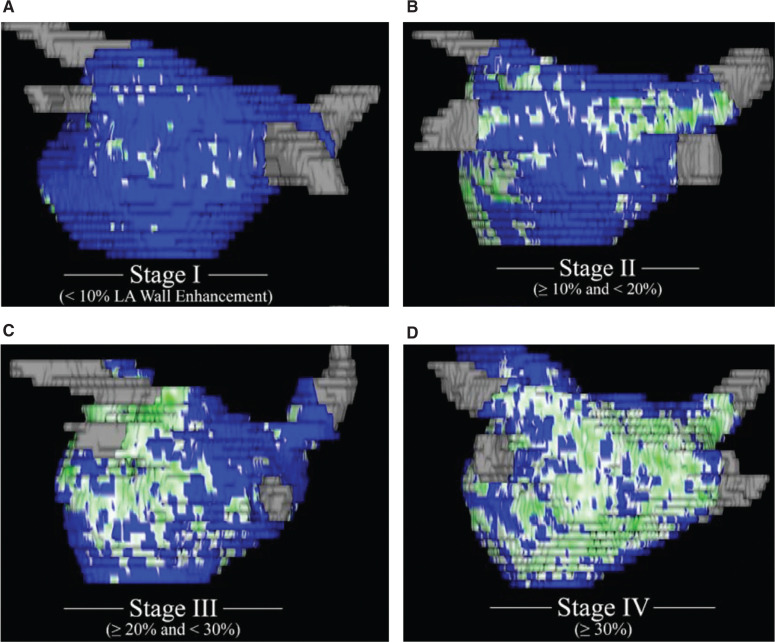
Progression of fibrosis in the posterior LA in patients with PRAF as confirmed by 3D delayed-enhancement magnetic resonance imaging scan. **A:** Stage 1 (< 10% of the atrial wall). **B:** Stage 2 (≥ 10% to < 20% of the atrial wall). **C:** Stage 3 (≥ 20% to < 30% of the atrial wall). **D:** Stage 4 (≥ 30% of the atrial wall).

Several recent anatomical studies including the Delayed-enhancement Magnetic Resonance Imaging Determinant of Successful Radiofrequency Catheter Ablation of AF (DECAAF) study showed that atrial remodeling or fibrosis usually extends beyond the PVs, with extensive fibrosis noted mostly in the posterior LA, suggesting a potential vulnerable substrate in the posterior LA beyond just the PVs.^10^ This appears to be a significant reason for why PVI itself is not adequate in managing the majority of patients with PRAF. **Figure 2** demonstrates the progression of posterior LA fibrosis as confirmed by three-dimensional (3D) delayed-enhancement magnetic resonance imaging scans.^10^

### Electrophysiological mechanisms in the posterior left atrium contributing to the progression of atrial fibrillation

The LA posterior wall often contains electrophysiological substrate(s) required for the maintenance of PRAF. The PVs and posterior LA (PV region) have been shown to sustain spontaneous and induced AF.^11^ Microreentrant and macroreentrant rotors important for AF perpetuation were observed in the posterior wall of the LA in animal studies.^12^ Furthermore, in animal studies using Langendorff-perfused sheep hearts, wherein 35 AF episodes were analyzed, the highest dominant frequency was most often (80%) localized to the posterior LA, near the PV ostium.^13,14^

## Ablation of the posterior left atrium and pulmonary vein region

### Ablation with endocardial box lesion set

After completion or achievement of PVI, a line of ablation lesions is created anteriorly across the LA roof by connecting the two superior PVs (roofline). Another line of ablation lesions is then created along the endocardial posterior LA to connect the left and right inferior PVs to isolate the LA posterior wall between the PVIs on both sides. Electrical isolation of the posterior wall was defined as pacing from the posterior wall with posterior wall capture and exit block to the remainder of the LA and/or the appearance of a dissociated potential along the linear ablation lines on the roof and posterior wall.

However, the success of endocardial posterior wall isolation along with PVI has been modest. A long-term evaluation of 27 patients with PRAF who underwent successful isolation of the PVs and a posterior wall box lesion set (wide antral PVI with subsequent connection by roof and floor lines) was reported. At 21 months of follow-up, 63% of the patients maintained sinus rhythm without antiarrhythmic drugs.^15^

Kottkamp et al.^16^ reported higher success rates with box isolation of fibrotic areas (BIFA) in patients with PRAF. The procedure, in addition to PVI, involved circumferential isolation of the fibrotic posterior LA (PV confluent low-voltage zones) identified by point-by-point voltage mapping. In a subgroup of patients with large areas of confluent fibrotic posterior LA, BIFA ablation single-procedure success rate was 72% at one year after surgery.

### Limitations of the endocardial ablation lesion sets to isolate the posterior left atrium

Completing a successful endocardial posterior box lesion set is difficult. Placement of a quality lesion with a durable posterior wall box lesion set using radiofrequency catheter ablation is challenging. Acute procedural success of the endocardial box lesion set may be limited by tissue edema that develops during the procedure, making the damage reversible on the subsidence of edema and inflammation; thus, the lesions may not be durable. Furthermore, because of the proximity of the esophagus to the posterior LA and the potential for an increase in esophageal temperatures, the posterior box lesion sets are left incomplete on several occasions. Nevertheless, it should be noted that, even with incomplete lines, sites containing the driver mechanisms in the posterior LA may have been coincidentally interrupted if they were in the path of incomplete lines.

Notably, advances in 3D mapping technologies including contact force-sensing ablation catheters and high-density mapping systems can address these limitations. Contact force-sensing catheters such as the SmartTouch^®^ catheter (Biosense Webster, Diamond Bar, CA, USA) and the TactiCath^®^ Quartz catheter (Abbott Laboratories, Chicago, IL, USA) use a force-sensing system to measure how much pressure is applied by the catheter tip to the heart tissue in real time. Adequate tissue contact, in addition to power delivery and catheter stability, will help to achieve durable, transmural lesions and to prevent complications resulting from the application of excessive force, especially when performing ablation in the posterior LA wall. Other advances in mapping system technologies such as multielectrode mapping catheters (PentaRay^®^; Biosense Webster, Diamond Bar, CA, USA) allow for the creation of high-density electrophysiology maps (eg, voltage and activation maps) within a short time and help in more easily localizing the gaps within the incomplete linear ablation lesions in the posterior wall, which can then be completed more efficiently. Esophageal deflection techniques using a transesophageal echocardiography probe or esophageal intraluminal balloon device can also assist in the avoidance of the delivery of ablation lesions in close proximity to the esophagus.

### Surgical ablation of the left atrium

The Cox maze III procedure, or the so-called cut-and-sew maze procedure, was introduced in 1987.^17^ Isolated, nonrandomized, and noncontrolled studies have reported freedom from AF rates after this procedure of higher than 90%.^18^ The Cox maze IV procedure improved upon these outcomes by using custom devices that ablate tissue using radiofrequency or cryoablation versus the incisions of the original maze procedure. This technique has been shown to be equally efficacious in single-center reports.^19^ A recent systematic review examining both the Cox maze III and IV techniques reported that freedom from AF rates in patients with PRAF were lower, but still excellent, at ~78% and 84%, respectively.^20^ However, due to its complexity and significant associated morbidity, the use of surgical ablation is typically confined mainly to concomitant procedures.

In an attempt to reduce the surgical morbidity of the procedure and eliminate the need for cardiopulmonary bypass, a minimally invasive thoracoscopic approach has been developed. Stand-alone minimally invasive ablation in patients with paroxysmal AF results in a freedom from AF rate of 91% at one year. However, in patients with PRAF, the stand-alone epicardial surgical ablation has a similar rate of success to those of percutaneous ablation approaches, with a freedom from AF rate at six months of ~53%.^21^

Challenges for the epicardial approach alone include an inability to confirm entry/exit block; an inadequacy of PVI, particularly in the posterior LA; and the mitral and cavotricuspid isthmus not being fully reachable from an epicardial approach. The AF Catheter Ablation versus Surgical Ablation Treatment (FAST) trial compared the efficacy and safety of surgical ablation with that of catheter ablation and found that, although surgical ablation had a higher success at 12 months (65.6% versus 36.5%), adverse events were also significantly higher in the surgical ablation group (34.4%).^22^

### Hybrid ablation of the pulmonary vein region (endocardial pulmonary vein isolation and epicardial posterior left atrial substrate ablation)

Because of the significant morbidity of extensive surgical ablation and the limited efficacy of endocardial ablation in patients with PRAF and LS-PRAF, the hybrid approach was developed, which involves the combination of an epicardial approach by a surgeon and a percutaneous endocardial approach by an electrophysiologist. The epicardial and endocardial ablation approaches are complementary. Combined simultaneous thoracoscopic surgical ablation with posterior LA box lesion set and transvenous catheter AF ablation was noted to be safe and feasible.^23^ This approach of thoracoscopic posterior box lesion followed by electrophysiological evaluation and endocardial ablation at 30 days with monitoring using a continuous rhythm monitoring device was shown to be durable, with 77.7% of patients maintaining sinus rhythm at a mean follow-up time of 30 months.

Other centers have investigated combined surgical and catheter ablation, with early mixed results. Gelsomino et al.^24^ conducted a systematic literature review on the efficacy and safety of hybrid AF ablation and found success rates (freedom from AF and antiarrhythmic drugs at six or 12 months) of 36.8% to 88.9%.

Recently, a minimally invasive subxiphoid approach for epicardial posterior AF ablation was developed. A hybrid ablation approach involving this subxiphoid epicardial posterior AF ablation approach in combination with endocardial AF ablation was shown to be safe and effective with less AF recurrence, fewer redo ablation procedures, and with an AF-free survival rate of 72% versus 51% (p = 0.01).^25^

## Our approach and experience with hybrid staged pulmonary vein region ablation

### Epicardial posterior left atrial wall ablation

The surgeon performs posterior LA wall epicardial ablation using a minimally invasive, subxiphoid, supradiaphragmatic 2-cm to 3-cm incision. This subxiphoid, supradiaphragmatic approach reduces the potential risk of hepatic laceration or injury. The lower inferior part of the pericardium is opened, and a thoracoscope is inserted over the guide, with identification of the PVs and the coronary sinus performed. Prior to the start of the case, the presence of thrombus in the LA and LA appendage is ruled out using transesophageal echocardiography.

Intrapericardial access is achieved using minimally invasive surgical techniques with the use of the endoscope and minimally invasive tools. A 3-cm EPi-Sense^®^-AF Guided Coagulation System with VisiTrax^®^ (AtriCure, Inc., Mason, OH, USA), which has saline irrigation and a vacuum attached, is then inserted and between 35 and 45 lesions (depending on the size of the LA and pericardial reflection anatomy) are created in the posterior LA from the left PV ostial region to the right PV ostial region, while the inferior border is marked by the coronary sinus. Each lesion is performed at 30 W for 90 seconds with monitoring for adequate impedance drop as well as direct tissue visualization via thoracoscopy. During and after each lesion is created, room-temperature saline is used to irrigate for cooling and to prevent collateral injury. Esophageal temperature is monitored via a previously inserted esophageal temperature probe located adjacent to the posterior LA under fluoroscopy. Ablation is stopped for any increase in esophageal temperature of > 0.5°C from the baseline temperature and saline irrigation is performed as needed. Special care is taken to avoid the phrenic nerve region by accessing the pericardium posterior to the phrenic nerve using devices that do not produce tension or lateral thermal spread. Hemodynamics are continuously monitored with an arterial line during the entire procedure/surgery. After ablation is completed, a 24-French soft BLAKE drain (Ethicon, Somerville, NJ, USA) is left in the pericardial space for two days and is removed before the patient is discharged home. The average hospital stay for this procedure is two days to three days. Postoperatively, patients will be continued on the anti-inflammatory medicine colchicine for two weeks after the procedure. Anticoagulation is not interrupted for this surgery/procedure, and the surgery is performed on therapeutic anticoagulation.

### Endocardial ablation

**Figure 3:**
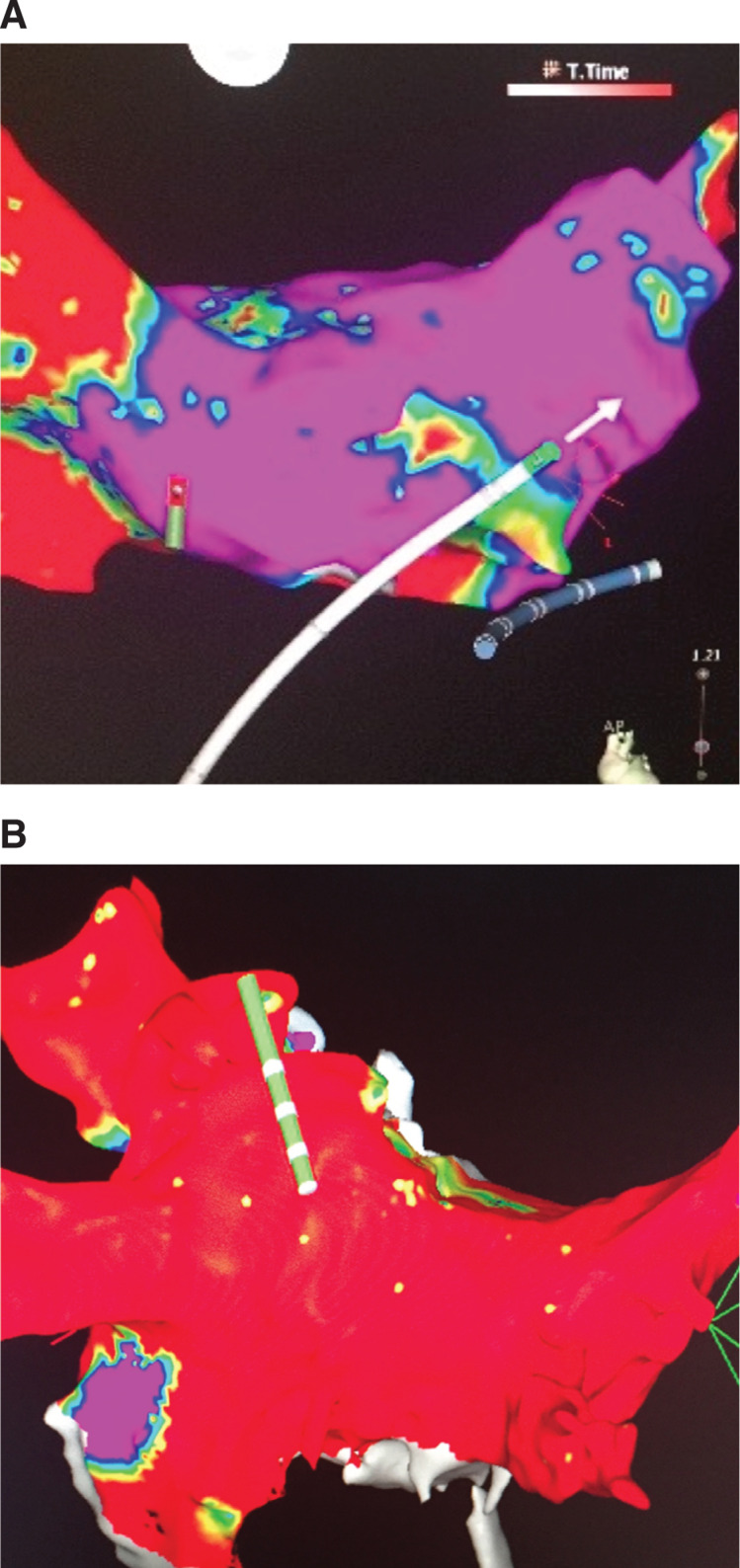
Demonstration of debulking of the posterior LA (PV region) on 3D map with loss of voltage on 3D map after hybrid epicardial and endocardial AF ablation. **A:** Before ablation. **B:** After ablation (red coloring indicates voltage < 0.3 mV).

At between five weeks and six weeks following epicardial AF ablation, patients undergo endocardial AF ablation. After obtaining bilateral femoral vein access, standard diagnostic catheters are positioned including the quadripolar catheter for His recording and a multipolar catheter for coronary sinus recording and pacing. Intravenous heparin bolus is administered prior to transseptal puncture. Under intracardiac echocardiography guidance, transseptal puncture is performed using a long sheath and a transseptal needle. Additional heparin bolus is administered and maintained on a heparin drip to achieve and maintain an activated clotting time of between 300 seconds and 350 seconds throughout the procedure. An endocardial map of the posterior LA and proximal PV trunks is created using the CARTO^®^ 3D mapping system (Biosense Webster, Diamond Bar, CA, USA). A voltage map of the posterior LA is created to assess the completeness of the prior epicardial LA posterior wall ablation **(Figure 3)**. PVI is performed with radiofrequency catheter ablation. PVI is confirmed by demonstrating exit and entrance block by pacing from the LA appendage and within all PVs. In patients with incomplete posterior epicardial AF ablation, additional radiofrequency ablation can be performed using an 8-French, 3.5-mm irrigated-tip radiofrequency catheter on the posterior wall with 25 W of energy applied for 30 seconds at each lesion site to homogenize scar in the posterior LA **(Figure 3)**. This is done while closely monitoring temperature changes with an esophageal temperature probe in close proximity to the ablation lesions in the posterior LA. Ablation is stopped if an increase in esophageal temperature of > 0.5°C with radiofrequency energy ablation occurs. In cases of incomplete or difficult LA posterior wall scar homogenization, an irrigated-tip radiofrequency catheter is used to create a line of ablation lesions superiorly and anteriorly across the LA roof with 25 W of power by connecting the bilateral superior PVI lesion sets. Block across the roofline is confirmed with activation mapping as well as with differential pacing. Postablation, the patient is restarted on their previous oral anticoagulation regimen after the sheaths are pulled and adequate hemostasis is achieved at the access sites.

The advantages of the staged epicardial and endocardial AF ablation approach include (1) a shorter time under anesthesia; (2) the surgeon and electrophysiologist working in their respective familiar environments; and (3) the electrophysiologist working on a healed substrate without tissue edema. The main disadvantage is the requirement of a two-day hospital stay, at minimum, and the potential for the patient to not return for the second stage of the procedure.

## Results

From our medical center, we have at this time three-month follow-up data on seven patients with LS-PRAF **(Table 2)** who underwent convergent or hybrid AF ablation. Of these, three patients successfully completed a six-month follow-up. The mean age of the patients was 52.6 years ± 11.2 years. These patients had previously failed multiple antiarrhythmic drugs (seven patients), cardioversion (seven patients), and/or AF ablation (four patients). Additionally, all of these patients underwent epicardial posterior LA ablation followed by endocardial PVI at five weeks to six weeks later as well as testing of the posterior LA ablation performed previously, and, if needed, additional ablation for the homogenization of posterior LA scar. All patients were discharged and were continued on previously failed antiarrhythmic drug therapy (one patient on class 1C flecainide, four patients on class III sotalol, and two patients on class III dofetilide). All of these patients (7/7; 100%) were followed for at least three months, and three of the seven (43%) patients participated in follow-up for at least six months. All seven patients showed a maintenance of sinus rhythm at their latest follow-up visit. One patient showed pericarditis and a small pericardial effusion after epicardial AF ablation that resolved spontaneously. No other major complications were noted.

### Case presentations

The following are case examples of patients with prior failed endocardial AF ablations who then underwent hybrid AF ablation.

**Figure 4:**
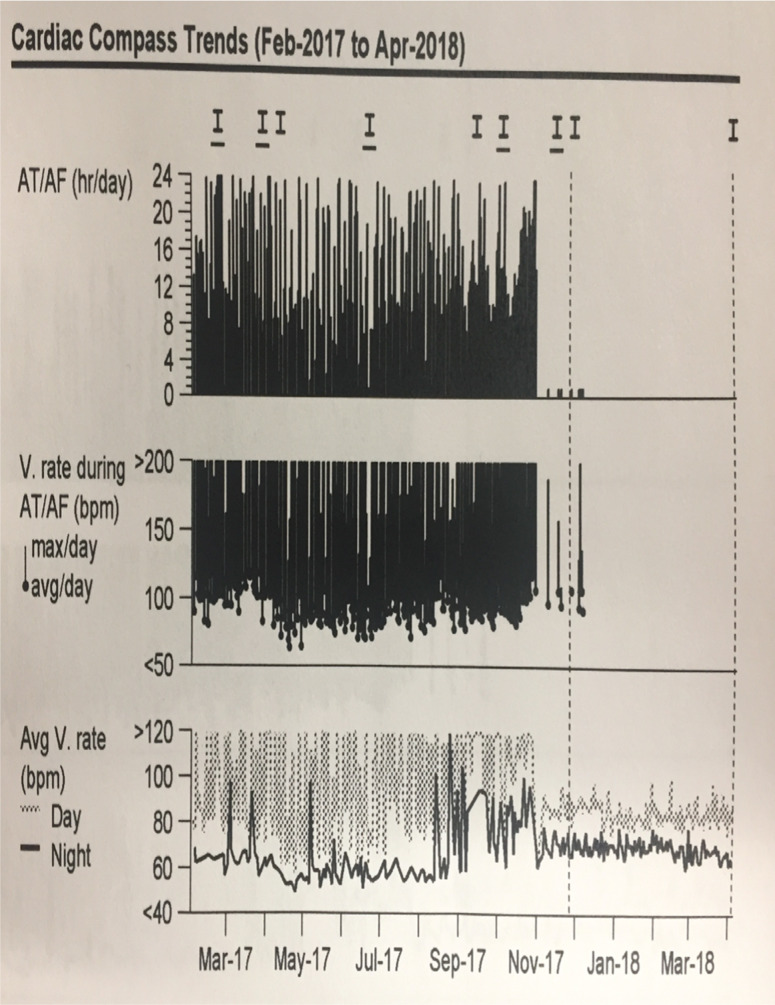
AF burden reduction following hybrid ablation of the posterior LA (PV region).

***Case 1.*** A 55-year-old male with a history of obesity (body mass index: 37.65 kg/m^2^), obstructive sleep apnea, hypertension, and diabetes mellitus with LS-PRAF who underwent multiple failed cardioversions and two failed catheter ablations (ie, PVI with radiofrequency ablation in 2013; PVI and LA mitral isthmus ablation as well as right atrial cavotricuspid isthmus ablation using radiofrequency ablation in 2014) presented for further evaluation. The patient had early recurrence of symptomatic AF with increasing AF burden (60% to 70%) on a previously implanted loop recorder. A recent computed tomography angiogram chest scan demonstrated a severely enlarged LA and no PV stenosis from the prior ablations. The patient subsequently underwent hybrid epicardial AF ablation (on August 23, 2017) and developed a small pericardial effusion postsurgery that did not require drainage. Six weeks later, he showed resolution of the pericardial effusion. He then underwent endocardial AF ablation (on October 31, 2017), with testing of the epicardial posterior wall lesions and homogenization of the posterior LA scar as well as completion of PVI by disconnecting the reconnected PV. The patient showed no further significant AF recurrences and a near-absent AF burden (0.1%) on his previously implanted loop recorder at six months after endocardial ablation **(Figure 4)**.

***Case 2.*** A 46-year-old female with a history of obesity (body mass index: 46.94 kg/m^2^), obstructive sleep apnea, diabetes mellitus, and diastolic heart failure with LS-PRAF who underwent multiple failed cardioversions presented to the clinic. She had no success with multiple antiarrhythmic drugs (eg, sotalol, dronedarone, dofetilide) and showed no success following AF ablation (PVI and CFAE ablation in 2013), with recurrence of symptomatic AF with increasing AF burden in last two years with failed repeated cardioversions. A computed tomography angriogram chest scan showed severe LA enlargement and no PV stenosis from the prior ablation. She then underwent hybrid epicardial AF ablation (on October 4, 2017) and had no complications postsurgery. After six weeks, she underwent endocardial AF ablation (on November 16, 2017) with testing of the epicardial posterior wall lesions and completion of posterior LA wall scar homogenization and PVI (she needed circumferential left-sided PVI). The patient had a recent follow-up electrocardiogram performed at six months after endocardial ablation that indicated no further symptomatic AF recurrences and the continued maintenance of sinus rhythm as has been documented on four serial electrocardiograms thus far.

## Discussion

The current endocardial ablation strategies for PRAF and LS-PRAF have limited efficacy with less favorable outcomes and high recurrence rates than those observed in patients with PVI alone. Additional ablation strategies have included linear ablation lesions, ablation of CFAEs, autonomic ganglionic plexi ablation, and “rotor” ablation, with no reproducible results of improved efficacy **(Table 1)**. This led to revisiting our understanding of the electrophysiological mechanisms for the development and progression of PRAF and LS-PRAF and the creation of effective hybrid nonpharmacologic management strategies.

The LA posterior wall (PV region) is important in the initiation and maintenance of AF for multiple reasons. Embryologically, the LA posterior wall between the PVs has the same embryologic origin as the PVs and therefore may share a similar propensity for housing triggers and also perpetuating AF. The PV region including the posterior LA wall contains favorable conditions for reentry. Complex fiber orientation within the PV region and posterior LA wall creates anisotropy, allowing for conduction block, reentry, and wave break.^26^ The PV region and posterior LA wall are subject to increased wall stress as compared with other areas of the atria.^27^ Histopathological examination in LS-PRAF due to mitral valve disease revealed greater myocytolysis and interstitial changes in the posterior LA wall.^28^ This fibrosis in the posterior LA wall promotes slow conduction and reentry and may provide stabilizing anchor points for spiral waves. Consequently, rotors, focal sources of AF, and important CFAE s are often localized to the PV region and posterior LA wall.^29,30^

Electrical isolation of the PV region and posterior LA wall (by combined hybrid LA endocardial and posterior LA epicardial ablation) would eliminate these triggers and major drivers of AF while debulking the atria, leaving less remaining available substrates for fibrillation. There is potentially a reduced impact on the mechanical function of the LA with debulking of the posterior LA. The posterior LA is predominantly a receiving chamber and has very little mechanical or contractile function, which is predominantly contributed to by the anterior LA and/or LA appendage. The recently reported experience with hybrid epicardial and endocardial ablation of the posterior LA (PV region) appears to be promising, with improved efficacy **(Table 1)**. This was reflected by our experience with hybrid posterior LA (PV region) ablation with 100% efficacy at three months’ follow-up.

### Limitations

The major limitation of our experience is our inclusion of very few patients with a limited follow-up period (three months). Furthermore, the long-term consequences of the debulking of the posterior LA are not completely known at this time. With the ongoing advancements in 3D mapping (eg, multielectrode high-density mapping) and ablation (eg, contact-force sensing) technologies as well as in esophageal deflection techniques, posterior LA wall isolation can potentially be safely and efficiently achieved by endocardial ablation alone. Future large clinical trials of combined PVI and posterior wall isolation by endocardial technique alone or via hybrid AF ablation of the posterior LA along with PVI (PV region ablation) with longer-term follow-up will potentially contribute to clarifying and addressing these issues.

## Figures and Tables

**Table 1:** Ablation Strategies and Success Rates for PRAF and LS-PRAF Patients Based on the Current Proposed Mechanisms

Ablation Strategies	Targets		Potential Substrate(s) Altered		Endpoints During Ablation		Efficacy Outcome(s) (% Maintenance of Sinus Rhythm)	
PV isolation	•	PV antrum and encircling tissue	•	PV arrhythmogenicity autonomics	•	Complete PV and antral electrical isolation	•	Endocardial ablation by Chao et al.^4^ ∘ Single procedure: 28.4% at three years ∘ Multiple procedures: 51.1% at three years
			•	Microreentry			•	STAR AF II study^6^ PVI arm ∘ 59% at 18 months
PVI + linear ablation	•	LA roof mitral isthmus	•	Macroreentry with or without autonomics	•	Conduction block across lines	•	STAR AF II study^6^ PVI + LAL ablation arm ∘ 46% at 18 months
	•	Posterior wall isolation	•	Rotors			•	Hamburg sequential ablation strategy^5^ PVI + LAL for LS-PRAF ∘ 20% at five years (after single procedure) ∘ 45% at five years (after multiple procedures)
PVI + electrogram-guided ablation	•	CFAEs	•	Rotors	•	AF termination	•	STAR AF II study^6^ PVI + CFAE ablation arm ∘ 48% at 18 months
	•	Frequency gradient	•	High-frequency sources	•	Elimination of CFAEs		
	•	Activation gradient						
Ganglionated plexi/autonomic ablation	•	Parasympathetic ganglionated plexi LOM	•	Autonomics	•	Absence of vagal response	•	Pokushalov et al.^7^ ∘ 38.2% at 24 months ∘ 59.6% with repeat circumferential PVI ablation
PVI + endocardial circumferential ablation of posterior low-voltage zone	•	Fibrotic posterior LA	•	PV confluent low-voltage zones	•	Complete PV and posterior low-voltage zone (scar) electrical isolation	•	Kottkamp et al.^16^ PVI + boxed isolation of fibrotic posterior LA (BIFA ablation) ∘ 72% at one-year follow-up
Hybrid/convergent AF ablation (endocardial PVI + minimally invasive epicardial posterior LA ablation)	•	PV region (PVs and posterior LA)	•	PV arrhythmogenicity	•	Complete PV and posterior LA (PV region) electrical isolation	•	Kress et al.^25^ hybrid epicardial and endocardial posterior LA ablation ∘ 72% at 16 months
			•	Posterior LA or PV region isolation				

PRAF: persistent atrial fibrillation; LS-PRAF: longstanding persistent atrial fibrillation; PV: pulmonary vein; PVI: pulmonary vein isolation; AF: atrial fibrillation; STAR AF II: Substrate and Trigger Ablation for Reduction of AF Trial Part II; LA: left atrium; CFAE: complex fractionated atrial electrogram; BIFA: box isolation of fibrotic areas; LAL: left atrial linear ablation line; LOM: ligament of Marshall.

**Table 2:** Description of Patients Who Underwent Hybrid/Convergent AF Ablation

Patient Number	Age	Sex	BMI	AF Duration	LA Size (CT Scan Angiogram)	Concomitant AAD Therapy	Prior Failed Endocardial AF Ablations	Prior Failed Cardioversions	Monitoring After Ablation
1	55 years	M	37.7 kg/m^2^	10 years	6.6 cm × 4.1 cm	Dofetilide	Yes (4)	Yes (12)	AF burden on implantable loop recorder + serial ECGs
2	46 years	F	47.8 kg/m^2^	10 years	7.6 cm × 5.1 cm	Dofetilide	Yes (2)	Yes (3–4)	Serial ECGs
3	45 years	M	36.3 kg/m^2^	3 years	8.5 cm × 4.8 cm	Flecainide	Yes (1)	Yes (3)	Serial ECGs + 48-hour Holter monitor
4	67 years	M	25.0 kg/m^2^	5 years	8.1 cm × 5.1 cm	Sotalol	No	Yes (2)	Serial ECGs
5	39 years	M	35.9 kg/m^2^	3 years	6.8 cm × 3.6 cm	Sotalol	No	Yes (3)	Serial ECGs + 48-hour Holter monitor
6	50 years	M	30.4 kg/m^2^	2 years	10.1 cm × 5.7 cm	Sotalol	No	Yes (1)	Serial ECGs
7	66 years	M	30.1 kg/m^2^	10 years	7.1 cm × 5.9 cm	Sotalol	Yes (2)	Yes (3)	AF burden on pacemaker + serial ECGs

AF: atrial fibrillation; BMI: body mass index; LA: left atrium; CT: computed tomography; AAD: antiarrhythmic drug; M: male; F: female; ECG: electrocardiogram.
